# Peroxisome proliferator activated receptor alpha inhibits hepatocarcinogenesis through mediating NF-κB signaling pathway

**DOI:** 10.18632/oncotarget.2212

**Published:** 2014-07-13

**Authors:** Ning Zhang, Eagle S. H. Chu, Jingwan Zhang, Xiaoxing Li, Qiaoyi Liang, Jie Chen, Minhu Chen, Narci Teoh, Geoffrey Farrell, Joseph J.Y. Sung, Jun Yu

**Affiliations:** ^1^ Institute of Digestive Disease and Department of Medicine and Therapeutics, State Key Laboratory of Digestive Disease and Li Ka Shing Institute of Health Sciences, The Chinese University of Hong Kong, Hong Kong SAR, China; ^2^ Department of Gastroenterology, First Affiliated Hospital, Sun Yat-sen University, Guangzhou, China; ^3^ Gastrointestinal Cancer Biology & Therapeutics Laboratory, CUHK-Shenzhen Research Institute, Shenzhen, China; ^4^ Australian National University Medical School at The Canberra Hospital, Canberra, Australia

**Keywords:** PPARα, tumor suppressor, nuclear factor-kappa B, signaling pathway, hepatocellular carcinoma

## Abstract

Peroxisome proliferator-activated receptor alpha (PPARα) ligands have been reported to suppress cancer growth. However, the role of PPARα in hepatocarcinogenesis remains unclear. We investigated the functional significance of PPARα in hepatocellular carcinoma (HCC). PPARα-knockout (PPARα^-/-^) mice were more susceptible to diethylnitrosamine (DEN)-induced HCC at 6 months compared with wild-type (WT) littermates (80% *versus* 43%, *P* < 0.05). In resected HCCs, TUNEL-positive apoptotic cells were significantly less in PPARα^-/-^ mice than in WT mice (*P* < 0.01), commensurate with a reduction in cleaved caspase-3 and caspase-7 protein expression. Ki-67 staining showed increased cell proliferation in PPARα^-/-^ mice (*P* < 0.01), with concomitant up-regulation of cyclin-D1 and down-regulation of p15. Moreover, ectopic expression of PPARα in HCC cells significantly suppressed cell proliferation and induced apoptosis. The anti-tumorigenic function of PPARα was mediated via NF-κB as evidenced by inhibition of NF-κB promoter activity, diminution of phosphor-p65, phosphor-p50 and BCL2 levels, and enhancing IkBα protein. Chromatin immunoprecipitation analysis confirmed PPARα directly binds to the *IkBα* promoter. In conclusion, PPARα deficiency enhances susceptibility to DEN-initiated HCC. PPARα suppresses tumor cell growth by inhibiting cell proliferation and inducing cell apoptosis via direct targeting IκBα and NF-κB signaling pathway.

## INTRODUCTION

Hepatocellular carcinoma (HCC) is the fifth most common solid tumor worldwide and is the second leading cause of cancer-related deaths in China [1, 2]. Majority patients with HCC are diagnosed at an advanced stage with few curable options, and 5-year survival rates are less than 12% [3]. While those with small HCCs managed by surgical resection, the recurrence rate is up to 50% at 3 years [4]. Hence, there is pressing need for effective molecular targeted therapies for HCC.

Peroxisome proliferator-activated receptor alpha (PPARα) belongs to the nuclear hormone receptor superfamily and plays physiologic roles in energy homeostasis by modulating glucose and lipid metabolism, and transport [5]. PPARα expression has a major impact on the maintenance of mitochondrial beta-oxidation [6] and has been controversial whether it promotes, or suppresses cancer growth. Several reports describe PPARα agonists conferring inhibitory effects on cancer cell lines [7, 8] and animal models colorectal cancer, ovarian cancer and liver cancer [9-12] in a PPARα-dependent manner [13]. Further, PPARα activated by its agonists acts as a master regulator of inflammation, by suppressing interleukin (IL)-6, TNF-α and NF-κB in the liver [14,15]. The anti-inflammatory and anti-angiogenic effects of PPARα also promote the suppression of tumor growth by improving microenvironment. In light of these findings, we propose that PPARα may act as a potential tumor suppressor against hepatocarcinogenesis. However, HCC has been known to develop when PPARα agonists are administered long-term to rodents [16]. Thus, elucidating the role of PPARα whether acting as a tumor promoter or suppressor is important in understanding its contribution to liver carcinogenesis and may provide clues in developing effective treatments against this malignance.

To investigate the effect of PPARα in hepatocarcinogenesis, we injected PPARα deficient (PPARα^-/-^) and wild-type (WT) mice with diethylnitrosamine (DEN), a carcinogen that is commonly used to induce HCC [17]. Having noted that PPARα^-/-^ mice were remarkably sensitive to DEN-induced hepatocarcinogenesis, we examined the relationship of PPARα to the key regulators of cell proliferation and apoptosis in livers from these mice and investigated its tumor suppressive role as well as molecular bases by which PPARα exerts this function *in vitro.* We studied PPARα downstream effectors using cDNA expression array and confirmed that its direct target was NF-κB by ChIP-PCR. Our findings illustrate that PPARα is a potential tumor suppressor in HCC and raise the tantalizing possibility that PPARα has indeed therapeutic potential in treating this lethal cancer.

## RESULTS

### Deletion of PPARα accelerates DEN-induced liver carcinogenesis

**Figure 1:**
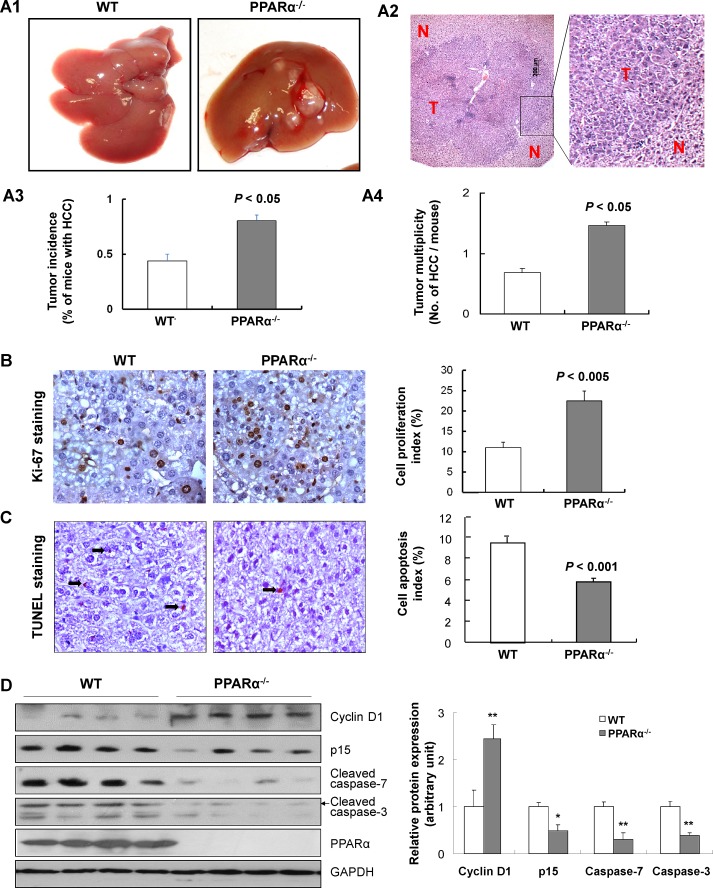
PPARα loss increases susceptibility to DEN-induced hepatocarcinogenesis (A1) Gross morphology of typical liver tumors from DEN-treated wild-type (WT) and PPARα knock out (PPARα^-/-^) male mice at 6 months. (A2) Representative microscopic features of HCC in H&E-stained liver sections of mice. T, tumor; N, adjacent non-tumor tissue. (A3) HCC incidence, and (A4) Number of HCCs per mouse in PPARα^-/-^ and WT mice at 6 months were counted and expressed as mean ± SD, 7-10 mice/group. (B) Cell proliferation in HCCs of PPARα^-/-^ and WT mice by Ki-67 immuno-staining (left panel). Quantitative assessment of cell proliferative index by Ki-67 positive cells (right panel). Data expressed as mean ± SD, 7-10 mice/group. (C) Apoptosis in HCCs of PPARα^-/-^ and WT mice by TUNEL staining (left panel). Quantitative assessment of apoptosis determined by TUNEL positive cells (right panel). Data are means ± SD, 7-10 mice/group. (D) Protein expression of cyclin D1, p15, cleaved caspase-7, cleaved caspase-3 and PPARα in HCCs from PPARα^-/-^ and WT mice determined by western blot. GAPDH was used as a loading control.

Fifteen WT mice and PPARα^-/-^ mice were injected with DEN and were examined at 6 and 8 months for the presence of liver tumors (Fig. 1A1). Liver tumors were confirmed as HCC by histology (Fig. 1A2). At 6 month, there was no significant difference in body or liver weights between the two genotypes (data not shown), nor in the macroscopic and histological features of hepatocytes between PPARα^-/-^ and WT animals. However, HCCs were identified in 43% of DEN-injected WT mice and more significantly in 80% of DEN-injected PPARα^-/-^ animals (*P* < 0.05) (Fig. 1A3). Moreover, tumor quantification was done by counting the macroscopic nodules. We found that the average number of tumors per animal was at least 2-fold higher in PPARα^-/-^ compared to WT mice (1.47 ± 0.29 *versus* 0.69 ± 0.24, *P* < 0.05) (Fig. 1A4). In contrast to WT mice, tumors were larger in PPARα^-/-^ mice, 42.8% of HCCs were larger than 2mm in DEN-injected PPARα^-/-^ mice compared to none in WT animals. Collectively, loss of PPARα appears to enhance DEN-induced hepatocarcinogenesis in mice.

### PPARα inhibits proliferation and induces apoptosis in hepatocarcinogenesis

Proliferative activity in HCCs from PPARα^-/-^ and WT mice was determined by Ki-67 immunostaining (Fig. 1B). HCCs from PPARα^-/-^ mice displayed significantly greater proliferative activity compared to their WT littermates (22.5 ± 2.4% versus 11.0 ± 1.3%, *P* < 0.005) (Fig. 1B). Macroscopic tumors from WT littermates and PPARα^-/-^ mice were dissected and used for protein extraction. We then analyzed expression of cell proliferation proteins by western blot, cyclin D1 and p15 in HCCs. Only low levels of cyclin D1 protein were detected in WT HCCs, while increased expression levels were detected in PPARα^-/-^ HCCs. In contrast, p15 protein was greatly decreased in PPARα^-/-^ HCCs compared to WT HCCs (Fig. 1D). Further, the apoptotic index was significantly reduced in HCCs from PPARα^-/-^ mice compared with WT mice, quantified by TUNEL positive cells (5.8% ± 0.4% versus 9.6% ± 0.5%, *P* < 0.001) (Fig. 1C). Moreover, apoptotic cell death, assessed by cleaved caspase-3 and cleaved caspase-7 in HCCs, was diminished in PPARα^-/-^ HCCs compared to WT HCCs (Fig. 1D). These findings indicate that the loss of PPARα in hepatocytes inhibits hepatocyte death and promotes subsequent proliferative activity in PPARα^-/-^ mice.

### PPARα is down-regulated in HCCs and plays a tumor suppressive role *in vitro*

**Figure 2:**
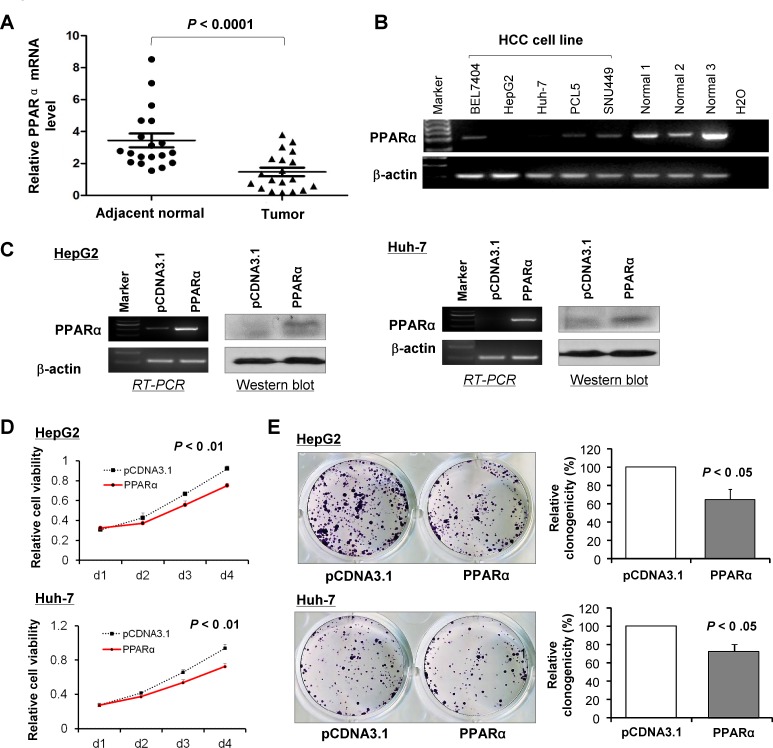
PPARα is down-regulated in human HCCs and cell lines and its expression suppresses proliferation of HCC cells *in vitro* (A) *PPARα* mRNA expression is significantly down-regulated in human HCCs compared with their adjacent non-tumor tissue (n = 27) by real-time PCR. (B) *PPARα* mRNA expression is silenced or down-regulated in human HCCs cell lines, but readily expressed in normal liver tissue samples. (C) Ectopic expression of PPARα mRNA and protein in HCC cell lines HepG2 and Huh-7 were evident by RT-PCR and western blot. (D) Ectopic expression of PPARα suppressed cell viability in HepG2 and Huh-7 cell lines by MTS assay. (E) PPARα inhibited colony formation in HepG2 and Huh-7 cells.

We examined the mRNA expression of *PPARα* in 27 paired tumor and adjacent normal primary HCC samples and found that *PPARα* was markedly down-regulated in tumor tissue compared with adjacent normal tissue (*P* < 0.001) (Fig. 2A). Downregulation or silence of *PPARα* expression was also observed in 5 HCC cell lines, relative to readily expression of *PPARα* in normal liver tissue samples (Fig. 2B). We further elucidated whether PPARα contributes to the growth inhibition of HCC cells. Two HCC cell lines, HepG2 and Huh-7, were stably transfected with PPARα/pcDNA3.1 or pcDNA3.1 plasmids. Ectopic expression of PPARα was confirmed in these two HCC cell lines by RT-PCR and western blot (Fig. 2C). Re-expression of PPARα caused a significant reduction in cell viability in HepG2 (*P* < 0.01) and in Huh-7 cells (*P* < 0.01) (Fig. 2D). The inhibitory effect on HCC cell growth was further confirmed by colony formation assay. There was 30% reduction in the colonies formed in HepG2 and Huh-7 cells with stable overexpression of PPARα compared with control cells (*P* < 0.05) (Fig. 2E). These results suggest that PPARα exhibits growth inhibitory activity in HCC cells.

### PPARα effectively induces HCC cell apoptosis *in vitro*

**Figure 3:**
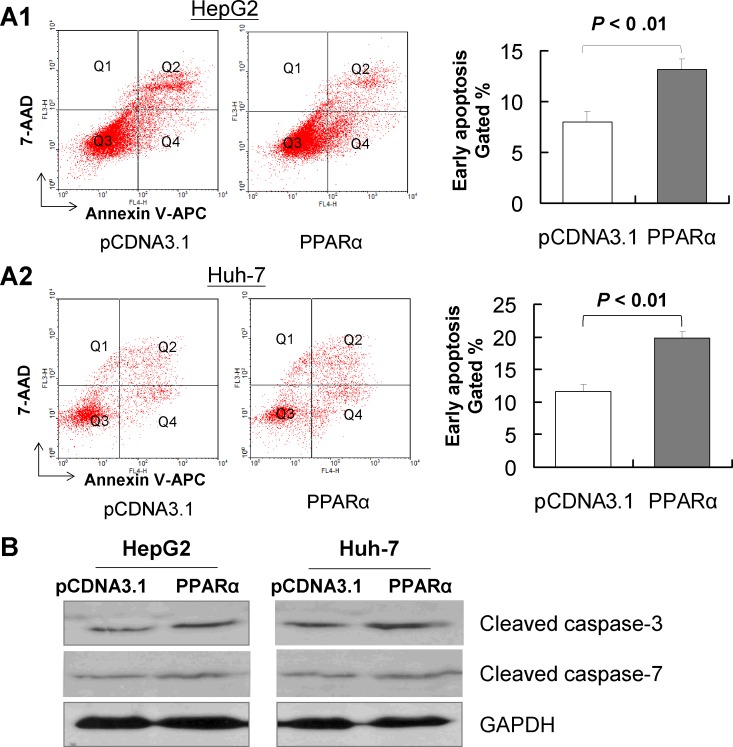
PPARα induces apoptosis of HCC cells *in vitro* Hep3B and Huh-7 cells were transfected with pcDNA3.1 or PPARα for 48 hours; the effect of PPARα overexpression on apoptosis was determined by FACS and annexin V. Apoptotic cells were significantly increased in PPARα-transfected cells compared with pcDNA3.1-transfected cells, in both HepG2 (A1) and in Huh-7 cell lines (A2). Results are mean ± SD from experiments performed in triplicate. (B) Protein expression of cleaved caspase-3 and cleaved caspase-7 in Hep3B and Huh-7 cells as evaluated by western blot. GAPDH was used as loading control.

In order to ascertain whether apoptosis was responsible for PPARα-induced growth inhibition *in vitro,* we performed flow cytometry with Annexin V and 7-AAD double staining in HepG2 and Huh7 cells. Our results showed that PPARα induced a significant increase in the number of early apoptotic cells in both HepG-2 (13.18 ± 0.30% *vs* 8.01 ± 0.34%, *P* < 0.01) and Huh-7 (19.83 ± 0.13% *vs* 11.66 ± 1.16%, *P* < 0.01) (Fig. 3A) compared with controls. The induction of cell apoptosis by PPARα was confirmed by the up-regulation of cleaved caspase-3 and cleaved caspase-7 (Fig. 3B).

### PPARα ablation results in dysregulation of gene expression profiles and signaling pathways in HCCs

**Figure 4:**
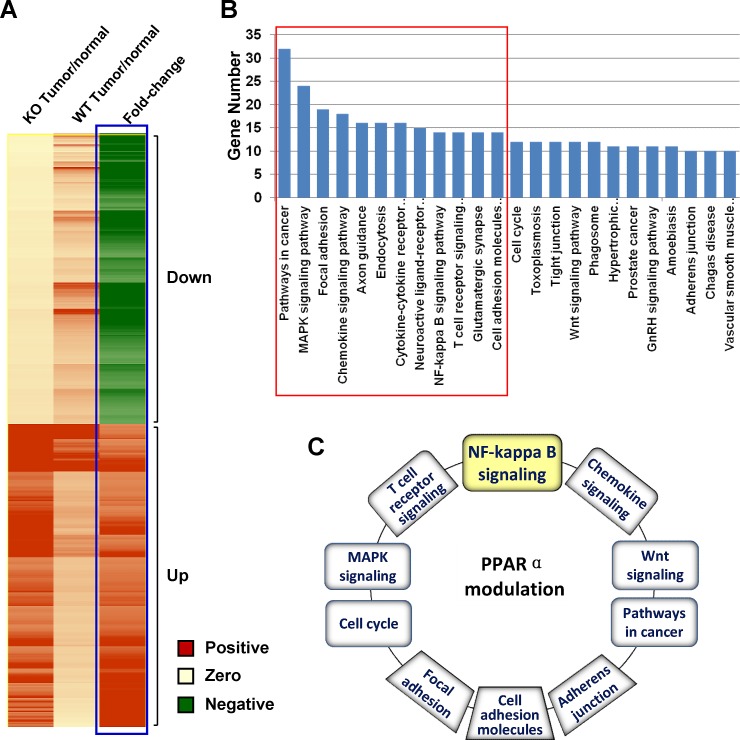
(A) Heatmap illustration of the differentially expressed genes in HCCs of PPARα^-/-^ and WT mice by gene expression microarray A total of 1,100 genes were differentially expressed in PPARα^-/-^ mice (tumor/normal) compared with those in WT mice (tumor/normal), with differential expression levels ≥ 2 (566 genes) or ≤ -2 (544 genes). (B) KEGG pathway enrichment analysis using GeneCodis (Genecodis version 2.0 http://genecodis.cnb.csic.es/). A total of 24 pathways were identified to be significantly dysregulated in PPARα^-/-^ mice compared with WT mice, with ≥10 genes involved and *P* < 0.05. (C) Ten pathways in PPARα^-/-^ mice had important functions associated with cell proliferation, cell apoptosis, migration, and cell-cycle regulation.

To understand the molecular mechanisms by which PPARα acts as a tumor suppressive factor in liver tumor development, gene expression microarray was performed in HCCs and non-HCC liver samples from DEN-treated PPARα^-/-^ and WT mice. A total of 1,100 genes were differentially expressed (fold-change ≥ 2) in PPARα^-/-^ mice (tumor/normal) compared with WT animals, in which 566 and 544 genes were up-regulated and down-regulated, respectively (Fig. 4A).

We further classified the PPARα-regulated gene candidates according to their biological functions by the Kyoto Encyclopedia of Genes and Genomes (KEGG) pathway analysis using GeneCodis. The results revealed that these genes were mainly enriched in 24 pathways (*P* < 0.05, ≥ 10 genes involved in each pathway) (Fig. 4B). Amongst them, 10 major pathways had important functions associated with cell proliferation, apoptosis, migration, and cell-cycle progression including pathways in cancer, MAPK signaling, NF-κB, T cell receptor and chemokine signaling, focal adhesion, adherens junction, cell adhesion molecules (CAMs), cell cycle and Wnt signaling pathway intermediates (*P* < 0.01) (Fig. 4C, S Table 1). Such data are consistent with previously identified functions of PPARα in diverse biological processes, which are indicative that the effects of PPARα inactivation on specific cellular pathways are relevant to cancer development.

### PPARα negatively regulates NF-κB signaling

**Figure 5:**
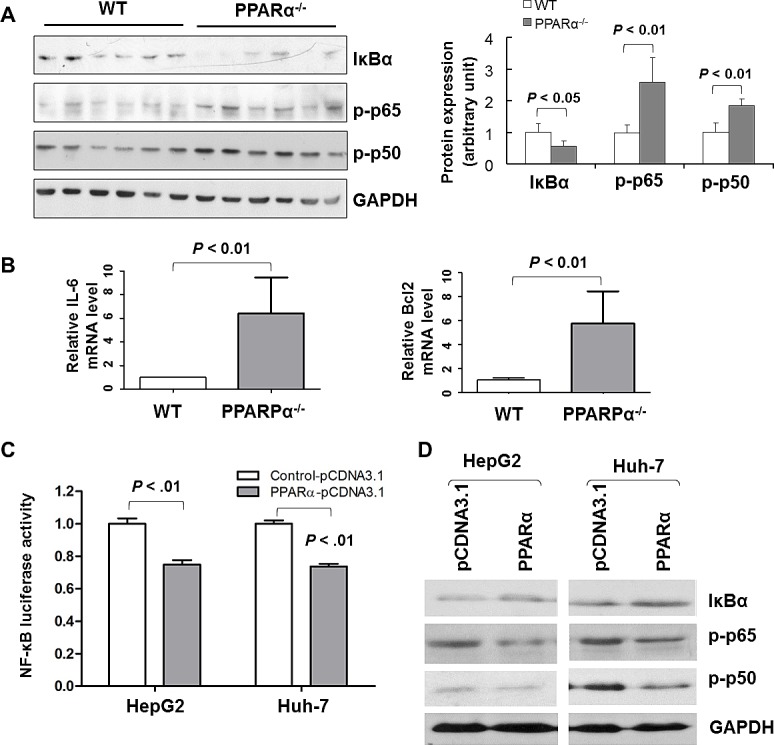
PPARα exerts inhibitory effects on NF-κB signaling *in vitro* (A) Protein expression of IκBα, phospho-NFκB p65 (p-p65) and phospho-NFκB p50 (p-p50) in HCC tissue from PPARα^-/-^ and WT mice by western blot. GAPDH was used as loading control. (B) mRNA expression of *Bcl2* and *IL-6* in HCC tissues of PPARα^-/-^ and WT mice by real-time PCR. β-actin was used as an internal control. (C) Luciferase activity was determined by dual luciferase activity assay at 48 hours post transfection with pcDNA3.1 or PPARα and luciferase plasmids in Hep3B and Huh-7 cells. PPARα significantly suppressed NF-κB-luc activity. Results expressed are means ± SD from six separate experiments. (D) Western blot evaluation of protein levels of IκBα, phospho-NFκB p65 (p-p65) and phospho-NFκB p50 (p-p50) in Hep3B and Huh-7 cells transfected with pcDNA3.1/PPARα or pcDNA3.1 control. GAPDH was used as loading control.

Notably, the NF-κB signaling pathway has been reported to be associated with PPARα [18]. We verified the importance of this pathway under regulation by PPARα in HCC by *in vivo* and *in vitro* experiments. As shown in Fig. 5A, IκBα protein expression, a super suppressor of NF-κB activation, was significantly decreased in HCCs of PPARα^-/-^ mice compared to tumors from WT mice (*P* < 0.05). In contrast, protein expression of the active forms of NF-κB, phospho-NF-κB p65 (*P* < 0.01) and phospho-NF-κB p50 (*P* < 0.01) were significantly enhanced in HCCs of PPARα^-/-^ mice. In keeping with these, NF-κB activator IL-6, and NF-κB downstream effector B-cell CLL/lymphoma 2 (Bcl2) were up-regulated in PPARα^-/-^ HCCs compared with WT HCCs (Fig. 5B).

To further confirm the interaction between the PPARα and NF-κB in HCC, we examined the functional effects of PPARα in NF-κB *in vitro* by using an NF-κB promoter luciferase reporter activity assay and western blot analysis. Transfection of PPARα significantly decreased NF-κB promoter luciferase reporter activity in both HepG2 (*P* < 0.01) and Huh-7 (*P* < 0.01) cells, compared with controls (Fig. 5C). Moreover, re-expression of PPARα induced IκBα, but repressed phospho-NFκB p65 and phospho-NFκB p50 protein expression in both HepG2 and Huh-7 cells by immunobloting (Fig. 5D).

### PPARα functionally interacts with IκBα

**Figure 6:**
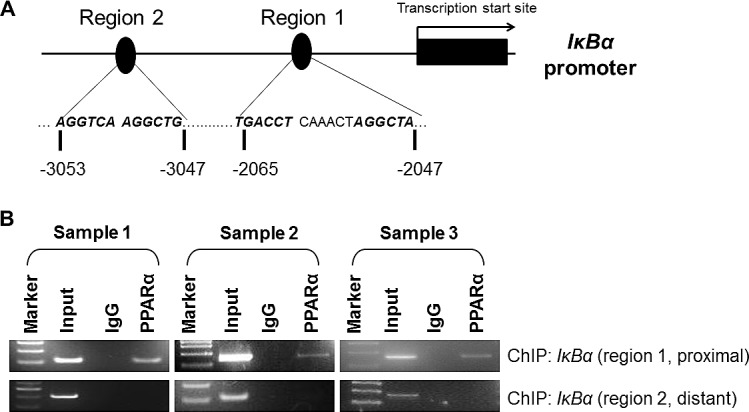
Functional interactions between PPARα and IκBα promoter (A) Computational prediction analyses indicate PPARα binding sites at the promoter region of IκBα gene with two potential regions of PPAR response element (5’-AGGTCA-3’). (B) ChIP-PCR assay reveals that PPARα binds to the promoter of *IκBα* gene at the proximal element, but not the distant element. Protein isolates from three individual WT mice livers were immunoprecipitated with anti-PPARα antibody. ChIP-PCR was subsequently performed to determine the recruitment of PPARα to the *IκBα* promoter regions.

To elucidate the key direct effectors of PPARα in NFκB signaling, we performed computational prediction of PPARα binding sites using MatInspector (Genomatix software gmbh, Munich, Germany). Only matrix and core similarity scores of > 0.8 were considered for the presence of PPARα binding sites. Interestingly, we identified two conserved regions in the promoter region of *IκBα* containing PPARα binding sites (5’-AGGTCA-3’) (Fig. 6A). For validation of direct interaction, we performed ChIP-PCR assay to determine whether PPARα can bind to the promoter of *IκBα* gene. Protein isolates from three individual WT mouse livers were immunoprecipitated with an anti-PPARα antibody. ChIP-PCR analysis revealed that PPARα indeed bound to the promoter region of *IκBα* (Fig. 6B), suggesting a direct protein-DNA interaction between PPARα and *IκBα*.

## DISCUSSION

Although activation of the PPARα by its agonists has been shown to inhibit cancer growth including HCC cell lines [7, 19], there have been no studies to mechanistically define the role of PPARα in hepatocarcinogenesis. In this study, we analyzed the role of PPARα in DEN-induced carcinogenesis using PPARα^-/-^ mice. WT as well as PPARα^-/-^ mice did not show spontaneous development of liver tumors up to the age of 6 months. However, in the DEN tumor induction model, loss of PPARα in mice resulted in a significant increase in HCC development compared to WT animals. Tumor multiplicity was also significantly increased in PPARα^-/-^ mice in comparison to WT mice. Moreover, we observed an increase in cell proliferation by Ki-67 staining and a reduction in cell death by TUNEL staining in the livers of PPARα^-/-^ mice. These results demonstrate that PPARα deletion induces a more aggressive phenotype of HCC. Thus, this is the first *in vivo* evidence for an essential role of PPARα in primary liver cancer development. We also found that PPARα was down-regulated in human HCCs compared with adjacent non-tumor tissue (*P* < 0.0001) and was silenced or down-regulated in all 5 HCC cell lines, further implying that PPARα may regulate HCC progression with a potential tumor suppressive property.

To further refine the functionality of PPARα in liver carcinogeness, we analyzed the effect of PPARα overexpression in two human HCC cell lines, HepG2 and Huh-7. Ectopic expression of PPARα led to a significant inhibition of HCC cell viability, and markedly reduced colony formation. Induction of apoptosis in both HepG2 and Huh-7 cells by PPARα was also observed, with concomitant the increase in protein expression of cleaved caspase-3 and cleaved caspase-7. Thus, it appears that the growth inhibitory effect of PPARα is associated with the suppression of cell proliferation and the induction of apoptosis that is mediated in part by activation of the caspase cascade. Our observations also concurrent with recent observations that activation of PPARα by the PPARα ligand, clofibrate, in HepG2 cells leads to notable reductions of cell numbers and an induction of apoptosis [19]. Collectively, these findings support an inhibitory role of PPARα in hepatocarcinogenesis.

To define the molecular basis of HCC development in the absence of PPARα, gene expression microarray analysis was performed to compare the expression profiles between the PPARα^-/-^ and WT HCCs. Among the 1,100 differentially expressed genes (fold-change ≥ 2) in PPARα^-/-^ mice, KEGG pathway analysis revealed that these candidate genes mainly enriched in 24 pathways, with 10 pathways important in cancer development such as pathways in cancer, MAPK, NF-κB, T cell receptor, chemokine signaling as wells as focal adhesion, adherens junction, cell adhesion molecules (CAMs), cell cycle and Wnt signaling pathway molecules (*P* < 0.01) (Fig. 4C, S Table 1), that regulate cell proliferation, apoptosis, cell-cycle, cell adhesion/metastasis, inflammation and signal transduction in tumors. Many pathways associated with MAPK, Wnt, focal adhesion, chemokine/T cell receptor and cell cycle signaling have been shown to be of significance in HCC [20-22]. These findings further support the functional role of PPARα in the development of liver cancer and provide a mechanism for the pro-HCC effects in the context of PPARα deletion.

The importance of NF-κB signaling activation has been well demonstrated in hepatocarcinogenesis [23-25]. The differential activation of NF-κB in HCCs from PPARα^-/-^ mice compared with WT animals may facilitate the cross-talk between hepatocyte and a tumor microenvironment that support tumor growth. NF-κB activity is tightly controlled at multiple levels by positive and negative regulatory elements. Increased levels of proinflammatory factors such as TNF (S Table 1) and cytokines (e.g. IL-6, Fig. 5B) observed in PPARα^-/-^ mice may plausibly play a role in the differential activation of NF-κB subunits, p65 and p50, in initiating a cascade of NF-κB activation. This is most likely achieved by phosphorylation and degradation of the IκB protein, a key suppressor of NF-κB. Indeed, our data demonstrate increased phosphorylation of p65 and p50, as well as decreased protein expression of IkBα in PPARα^-/-^ mice. Further, a direct interaction between PPARα and the promoter of IkBα was demonstrated by ChIP-PCR analysis, inferring that PPARα controls IκBα expression through direct PPARα protein-IκBα DNA interactions. A similar mechanism has been described by others, that PPARα inhibits NF-κB signaling via the induction of IκBα, although a direct interaction has not yet been clarified definitively between PPARα and IκBα [26-28].

Active PPAR*α* binding sites have been identified in the mouse *I*κ*B*α promoter region (-2,185 and -3,173 bp from transcriptional start site, TSS) and the human *I*κ*B*α promoter region (-1,000 and -1,795 bp from TSS) [28]. Thus, induction of *IκBα* is probably the key mechanism of NF-κB activation in the PPARα^-/-^ mice. Released NF-κB p65 and p50 translocate to the nucleus, where they are able to bind the promoter and enhancer regions containing κB sites, to mediate the transcription of related target genes such as Bcl2. Bcl2 harbors an NF-κB binding site in its promoter, which is responsible for the anti-apoptotic activity of NF-κB [29]. Using transient transfection assays, we confirmed that ectopic expression of PPARα decreases NF-κB promoter activity in two HCC cell lines, with concomitant down-regulatedion of p65 and p50 phosphorylation, and up-regulation of IκBα. These results further confirm that *PPARα* can transcriptionally induce IκBα expression which antagonizes NF-κB signaling, an observation which is consistent with the critical role for PPAR*α* in tumor suppression. We and others have previously reported that PPARα agonists negatively regulate NF-κB signaling in different cellular and animal models [15, 30, 31], implying that PPARα acts as a possible master inhibitor of NF-κB activation.

This study strengthens our understanding of how PPARα mediates hepatocytes proliferation in HCC and provides new mechanistic insights into the importance of PPARα in inhibiting liver cancer development; one such key pathway is via suppressing NF-κB activation. On the basis of these observations, we propose that strategies involving the enhancement of PPARα expression in the liver or in combination with current therapeutic regimens may potentially be effective in the management of HCC in human.

## MATERIALS AND METHODS

### Patients and tissues

Surgically excised HCCs, and surrounding non-tumorous liver tissues were obtained from 27 patients at Prince of Wales Hospital, The Chinese University of Hong Kong, Hong Kong. Written consent was obtained prior to surgical resection, and the study was approved by the Human Ethics Committee of The Chinese University of Hong Kong. Tissue collected was immediately snap-frozen and stored in liquid nitrogen for later analyses.

### Animals and experimental design

C57BL/6N PPARα^-/-^ mice were a gift by Professor Geoffrey Farrell (Australian National University Medical School at The Canberra Hospital, Canberra, Australia). Genotyping was performed by polymerase chain reaction (PCR). Male mice at 15 days of age received a single intraperitoneal injection of DEN (5 mg/kg body weight; Sigma Chemical Co., St. Louis, MO) to induce HCC. Mice were sacrificed at 6 months after injection. Livers were excised, weighed, and presence and dimensions of surface HCC nodules were measured. Tumors and non-tumorous liver tissue were removed and either fixed in 10% neutral-buffered formalin or snap frozen in liquid nitrogen. Liver sections from paraffin-embedded blocks were cut and H&E stained for histologic examination. All experiments in this study were approved by the Animal Experimentation Ethics Committee of the Chinese University of Hong Kong.

### Ki-67 staining

Ki-67 (Abcam, Cambridge, UK) was detected in paraffin-embedded liver sections of mice using an avidin-biotin complex immunoperoxidase method. The proliferation index was determined by counting the numbers of positive staining cells for Ki-67 as a percentage of the total number of cells. At least 1000 cells were counted each time.

### Terminal deoxynucleotidyl transferase-mediated nick-end labeling (TUNEL) staining

TUNEL staining was employed for detection of apoptosis in liver sections (Promega, Madison, WI). The apoptosis index was calculated as a percentage of TUNEL-positive cells showing unambiguous brown nuclear staining (n >1000).

### Human HCC cell lines and culture

The human HCC cell lines (BEL7404, Hep G2, Huh-7, PLC5, SNU449) were obtained from the American Type Culture Collection (ATCC, Manassas, VA). These cell lines were cultured in Dulbecco's modified Eagle medium with 10% fetal bovine serum (Gibco BRL, Rockville, MD), and maintained at 37°C in a humidified incubator with 5% CO_2_.

### Construction and transfection of PPARα expression vector

The entire coding sequence of human PPARα cDNA was cloned and inserted into the pcDNA3.1 vector. HCC cell lines Hep G2 and Huh-7 were transfected with pcDNA3.1-PPARα or pcDNA3.1 control plasmid using Lipofectamine 2000 (Invitrogen, Carlsbad, CA). Cells were collected for analyses at 48 h after transfection.

### Colony formation assay

After transfection, cells were collected and seeded (3×10^3^/well) in 6-well plates for 14 days. Colonies (≥ 50 cells/colony) were counted after fixed with 70% ethanol and stained with crystal violet solution.

### Cell viability assay

This was determined by 3-(4, 5-dimethylthiazol-2-yl)-5-(3-carboxymethoxyphenyl)-2-(4-sulfophenyl)-2H-tetrazolium (MTS) assay (Promega, Madison, WI). Briefly, 3 × 10^3^ cells/well was seeded in 96-well plates. 20 μl of reaction solution containing 333 μg/ml MTS and 25 μM phenazine ethosulfate was added to culture cells in 100 μl culture medium every 24 h and incubated at 37°C for 1 hours. Cell viability was measured as an optical value of the mixture (wave length of 490 nm).

### Cell apoptosis assay

Apoptosis was also measured by staining cells with Annexin V (APC-conjugated) and 7-amino-actinomycin (7-AAD) (BD Biosciences, San Jose, CA), then analyzed by fluorescence-activated cell sorting (FACScan, BD, Franklin Lakes, NJ). Cell populations were classified as viable (Annexin V-negative, 7-AAD-negative), early apoptotic (Annexin V-positive, 7-AAD-negative), late apoptotic (Annexin V-positive, 7-AAD-positive), or necrotic (Annexin V-negative, 7-AAD-positive).

### Western blot analysis

Total protein was extracted and protein concentration was measured by the method of DC protein assay Bradford (Bio-Rad, Hercules, CA). Thirty μg protein from each sample was separated on Tris-polyacrylamide gel by electrophoresis and blotted onto nitrocellulose membranes (GE Healthcare, Piscataway, NJ). Membranes were incubated with primary antibodies overnight at 4°C and an appropriate secondary antibody for 1 hour at room temperature. Primary antibodies used were: anti-cyclin D1 (sc-8396), anti-IκBα (sc-847), anti-phospho-NFκB p50 (sc-271908), anti-phospho-NFκB p65 (sc-8008) and anti-GAPDH (sc-365062) (Santa Cruz Biotechnology, Santa Cruz, CA); anti-p15 (ab53034) and anti-PPARα (ab97609) (Abcam); anti-Cleaved caspase-7 (#9491), anti-Cleaved caspase-3 (#9661) (Cell Signalling Technology, Danvers, MA). Proteins were visualized using ECL Plus Western blotting Detection Reagents (GE Healthcare, Piscataway, NJ).

### cDNA synthesis and RT-PCR

Total RNA was extracted from liver tissue and cell pellets using Qiazol reagent (Qiagen, Valencia, CA). Total RNA was reverse-transcribed by High Capacity cDNA Reverse Transcription Kit (Invitrogen). Semi-quantitative RT-PCR was performed using Hot-start DNA polymerase (Invitrogen). And real-time PCR was performed using SYBR Green master mixture (Applied Biosystems) on HT7900 system (Applied Biosystems, Foster City, CA).

### Gene expression microarray analysis

Gene expression profiles of HCCs and adjacent normal liver tissues from two pairs samples of PPARα^-/-^ and WT mice were analyzed using Whole Mouse Genome Microarray Kit, 4x44K (Agilent Technologies, Palo Alto, CA), which contained 43,379 cDNA clones. In brief, RNA was extracted using Qiazol reagent (Qiagen, Valencia, CA). The cDNA probes were prepared from 5 μg of total RNA labeled with Cy5-dUTP (red) or Cy3-dUTP (green) by reverse transcription (Amersham Biosciences, Piscataway, NJ). Two labeled cDNAs were competitively hybridized to the microarray. Signal intensities were analyzed using a GenePix 4000A scanner (Axon Instruments, Molecular Devices Crop., Palo Alto, CA). Array data were presented as log base 2 ratio of the Cy5/Cy3 signals. Gene expression patterns between HCCs and controls were analyzed using unsupervised hierarchical clustering.

### Dual-Luciferase reporter activity assay

Luciferase reporter activity of NF-кB-luc (5xNF-кB binding sites) was examined. HCC cells were co-transfected with NF-кB-luc (195 ng/ well), pRL-CMV vector (5 ng/ well), and pcDNA3.1 or PPARα plasmid (200 ng/ well). Cells were analysed at 48 hours after transfection, and the ratio of firefly to renilla luciferase activity was analyzed by the dual-luciferase reporter assay system (Promega).

### Chromatin immunoprecipitation (ChIP)

ChIP assay was performed on liver tissues from DEN-treated PPARα^-/-^ and WT mice using EZ-Magna ChIP A kit (Millipore, Billerica, MA). Chromatin DNA fragments were precipitated with 10 μg anti-PPARα antibody (ab97609) (Abcam, Cambridge, MA). DNA was then de-cross linked and extracted from the DNA-protein complex. Immunoprecipitated DNA was subjected to ChIP-PCR validation. Distant ChIP-PCR primers are as follows: 5’-GGCACAGTGTCGGACGATT-3’ (forward) and 5’-GCTTCAGTTTTCTCCTCATTGTCAA-3’ (reverse). The proximal primers are 5’-CCCAAGCGGAAGACAGATT-3’ (forward) and 5’-GTAAAGTATATGTTAACCCTTGGAA-3’ (reverse).

### Statistics

Statistical analysis was performed using the SPSS statistical software package (standard version 13.0, SPSS Inc., Chicago, IL). Data were expressed as mean ± standard deviation (SD) with *P* < 0.05 considered as statistically significant.

## SUPPLEMENTARY MATERIAL TABLE



## Abbreviations

Bcl2: B-cell CLL/lymphoma 2
cDNA: complementary DNA
ChIP: chromatin immunoprecipitation
DEN: diethylnitrosamine
ERK: extracellular signal regulated kinase
GAPDH: glyceraldehyde-3-phosphate dehydrogenase
HCC: hepatocellular carcinoma
H&E: hematoxylin and eosin
IL: interleukin
NF-κB: nuclear factor-kappa B
IκBα: NF-κB inhibitor alpha
JAK/STAT: Janus kinase/signal transducer and activator of transcription
MAPK: mitogen-activated protein kinase
PCR: polymerase chain reaction
PPARs: peroxisome proliferator-activated receptors
PPREs: peroxisome proliferator response elements
TUNEL: terminal deoxynucleotidyl transferase-mediated nick-end labelling
WT: wild-type
KO: knockout
PPARα^-/-^: PPARα-knockout
KEGG: Kyoto Encyclopedia of Genes and Genomes.
