# Supplementary material for: Peroxisome proliferator activated receptor alpha inhibits hepatocarcinogenesis through mediating NF-κB signaling pathway
Source: Oncotarget. 2014 Jul 13;5(18):8330–40. doi: 10.18632/oncotarget.2212 (PMC4226686; doi:10.18632/oncotarget.2212)
Supplement: Supplementary file 1 [file oncotarget-05-8330-s001.pdf]

Peroxisome Proliferator Activated Receptor Alpha Inhibits Hepatocarcinogenesis through Mediating NF-κB Signaling Pathway

Supplementary Material

S Table 1: Enrichment of Kyoto Encyclopedia of Genes and Genomes (KEGG) Pathways in Differentially Expressed Genes in PPARα$^{-/-}$ Tumor

**Pathways**	**NG** [a]	***P*** [b]	**Genes**	**Annotation**
Pathways in cancer	32/321	4.2E-07	Wnt9b,Hdac2,Ar,Bcl2,Fgfr2,Lama3,Igf1r,Lamc3,Nfkb1(p50),Wnt9a,Mecom,Tcf7,Fgf2,Fgf8,Cdkn1a,Mmp2,Cdkn2a,Egln3,Lama2,Rassf5,Bmp4,Mapk8,Ikbkg,Runx1t1,Il6,Jun,Cdh1,Tcf7l2,Fgfr1,Mmp9,Dvl2,Bax	Cancer related pathway
MAPK signaling	24/263	1.0E-04	Cacna1c,Fgfr2,Pak1,Cacnb1,Map2k6,Nfkb1(p50),Map3k3,Pla2g2c,Cacng6,Mecom,Fgf2,Fgf8,Cacng7,Nfatc2,Nfatc4,Bdnf,Cacna1e,Stk3,Cacna1b,Map3k8,Mapk8,Ikbkg,Jun,Fgfr1	Cell proliferation, differentiation and migration
NF-kB signaling	14/102	1.1E-04	Bcl2, Nfkb1(p50), Atm, Bcl2l1, Btk, Icam1, Ikbkg, Prkcb, Tnf, Tnfsf13b, Igh-VJ558, Malt1, Pias4, Ube2i	Immunity, inflammation and cell survival
T cell receptor	14/108	1.7E-04	Pak1,Ifng,Icos,Nfkb1(p50),Vav1,Il4,Cd28,Nfatc2,Nfatc4,Cd8a,Map3	Cell proliferation, cytokine

signaling			k8,Ikbkg,Fyn,Jun	production and differentiation
Chemokine signaling	18/177	2.0E-04	Gnai1,Pak1,Nfkb1(p50),Ccl7,Gng4,Vav1,Ccl3,Cxcl14,Pard3,Gng8,Ccl8,Elmo1,Prex1,Pf4,Ikbkg,Plcb1,Grk6,Gsk3a	Cellular activation, differentiation and survival
Focal adhesion	19/199	2.0E-04	Bcl2,Thbs4,Parvb,Lama3,Pak1,Igf1r,Lamc3,Tln2,Vav1,Pdpk1,Src,Col5a2,Itga7,Lama2,Mapk8,Fyn,Jun,Kdr,Thbs1	Cell motility, proliferation, Differentiation, and survival
Adherens junction	10/72	9.2E-04	Igf1r,Pvrl2,Tcf7,Src,Pard3,Fyn,Cdh1,Ctnnd1,Tcf7l2,Fgfr1	Cell movement and proliferation
Cell adhesion molecules	14/143	1.3E-03	Icosl,Icos,H2-T24,Cadm1,Selp,Pvrl2,Ncam2,Cd28,Cd8a,Cdh1,H2-Oa,Negr1,Ncam1,H2-M1	Cell–cell interactions
Cell cycle	12/123	3.4E-03	Hdac2,Chek2,Orc3,Stag2,Atm,Chek1,Atr,Cdkn1a,Cdkn2a,Cdc25a,Cdc23,Smc3	Cell cycle
Wnt signaling	12/150	8.3E-03	Wnt9b,Ppp2r1b,Wnt9a,Tcf7,Nfatc2,Nfatc4,Vangl2,Mapk8,Jun,Tcf7l2,Dvl2,Plcb1	Cell-fate specification, cell proliferation and division

[a]NG, number of annotated genes in the input list / number of annotated genes in the reference list.

[b]P value, corrected hypergeometric P values.
